# Local orthorhombic phase in zirconium oxide nanocrystals: insights from X-ray pair distribution function analysis

**DOI:** 10.1107/S1600576725001761

**Published:** 2025-04-04

**Authors:** Rohan Pokratath, Kumara Cordero-Edwards, Maryame Bina, Simon J. L. Billinge, Jonathan De Roo

**Affiliations:** a Department of Chemistry University of Basel Mattenstrasse 22 4058Basel Switzerland; b Catalan Institute of Nanoscience and Nanotechnology (ICN2), CSIC and BIST, Campus UAB, Bellaterra, 08193Barcelona, Spain; c Applied Physics and Applied Mathematics Department Columbia University New York NY USA; Australian Synchrotron, ANSTO, Australia

**Keywords:** local structure, nanocrystals, zirconium oxide, total scattering, pair distribution function, ferroelectrics

## Abstract

Zirconium dioxide (ZrO_2_) and hafnium dioxide (HfO_2_) have emerged as promising alternatives to conventional ferroelectric materials. Understanding the crystal phases of these oxides under different conditions is crucial for optimizing their properties. There are several theories for the (anti)ferroelectric properties; however, comprehensive analysis, particularly at the local structure level, is lacking. In this study, we investigate the local structure of ZrO_2_ nanocrystals using X-ray pair distribution function (PDF) analysis, revealing an unexpected local orthorhombic distortion irrespective of crystallite size. This finding suggests the potential existence of an intermediate orthorhombic phase during the microscopic switching pathway observed in previous studies. Additionally, we explore the influence of crystallite size and surface effects on the PDF. These results contribute to a deeper understanding of the structural dynamics in ZrO_2_ and offer insights for the design of next-generation ferroelectric materials.

## Abstract 2

The local structure of ZrO_2_ nanocrystals is investigated using X-ray pair distribution function analysis, revealing orthorhombic distortions independent of crystallite size. These findings suggest a potential intermediate phase in ferroelectric switching.

## 1. Introduction

Materials that exhibit switchable polarization have garnered significant interest due to their potential application in various fields, *e.g.* energy storage, energy harvesting, infrared sensors, high permittivity capacitors, ferroelectric random access memory, radio frequency identification and other optoelectronic devices (Okuno *et al.*, 2021 ▸; Böscke *et al.*, 2011 ▸; Park *et al.*, 2018 ▸; Wong & Salahuddin, 2015 ▸; Gurfinkel *et al.*, 2006 ▸; Lemey *et al.*, 2016 ▸; Wei *et al.*, 2022 ▸; Sharma *et al.*, 2021 ▸). Switchable polarization refers to the ability of certain materials to exhibit a reversible change in the orientation of their electric dipole moment under the application of an external electric field (ferroelectricity), pressure (piezoelectricity) or thermal variations (pyroelectricity). Since all ferroelectric materials are pyroelectric and piezoelectric, ferroelectricity is a unique behavior, which allows the interchange between electrical, mechanical and thermal energy. Standard ferroelectric materials, such as BaTiO_3_, are not compatible with silicon process technologies, leading to a demand for alternative materials. Since 2011, zirconium (ZrO_2_) and hafnium oxides (HfO_2_) have been considered as a potential replacement (Böscke *et al.*, 2011 ▸).

The crystal phase of both oxides varies with temperature and crystal size (Van den Eynden *et al.*, 2022 ▸). The monoclinic phase is favored under ambient conditions, transitioning to the tetragonal ZrO_2_/HfO_2_ phase at around 1170/1700°C and further transforming into the cubic fluorite-type phase at approximately 2370/2600°C. The tetragonal and cubic phases can also be stabilized at room temperature by reducing the crystal size, cation doping, oxygen vacancies, stress/strain, quenching after crystallization or modifying the surface energy (Garvie, 1978 ▸; Mikolajick & Schroeder, 2021 ▸). For ZrO_2_ the tetragonal phase becomes the stable form at room temperature when the crystal size falls below 30 nm, in agreement with the experimental results (Joo *et al.*, 2003 ▸; Depner *et al.*, 2009 ▸). There have also been reports of 20 nm tetragonal crystals (Robinson *et al.*, 2005 ▸). Conversely, for HfO_2_, the tetragonal phase becomes thermodynamically favored when the crystallite size falls below approximately 3.6 nm (Hunter *et al.*, 1979 ▸). This disparity underscores the distinct thermodynamic behavior of these materials.

Although both ZrO_2_ and HfO_2_ exhibit (anti)ferroelectric properties, the crystallographic explanations are often more explicitly provided for the latter (Mikolajick & Schroeder, 2021 ▸; Böscke *et al.*, 2011 ▸; Huan *et al.*, 2014 ▸; Müller *et al.*, 2012 ▸). Despite numerous reports on the (anti)ferroelectric properties in ZrO_2_ (Lin *et al.*, 2017 ▸; Pešić *et al.*, 2016 ▸; Reyes-Lillo *et al.*, 2014 ▸; Lombardo *et al.*, 2020 ▸; Silva *et al.*, 2023 ▸), there is a lack of comprehensive crystallographic analysis, especially of the local structure (Liu *et al.*, 2024 ▸). The (anti)ferroelectric polarization emerges from a transition from a non-polar to a polar phase. Hoffmann *et al.* (2022 ▸) observed the transition between the tetragonal and orthorhombic *Pca*2_1_ phase upon the application of an electric field. However, the microscopic switching pathway between the phases remains unclear, with the potential inclusion of an intermediate orthorhombic *Pmn*2_1_ phase (Hoffmann *et al.*, 2022 ▸). In this work, we investigate the local structure of ZrO_2_ nanocrystals with sizes ranging from 2.5 to 5.6 nm using X-ray pair distribution function (PDF) analysis. While the structure of these nanocrystals was initially presumed to be tetragonal, our analysis reveals an intriguing local orthorhombic *Pmn*2_1_ distortion regardless of crystallite size. This finding untangles the possibility of an intermediate orthorhombic *Pmn*2_1_ phase during the microscopic switching pathway observed by Hoffmann *et al.* (2022 ▸). Additionally, we investigate two factors that may influence the short-range PDF: crystallite size and surface effects.

## 2. Results and discussion

### 2.1. Identifying the local distortion

**Figure 1:**
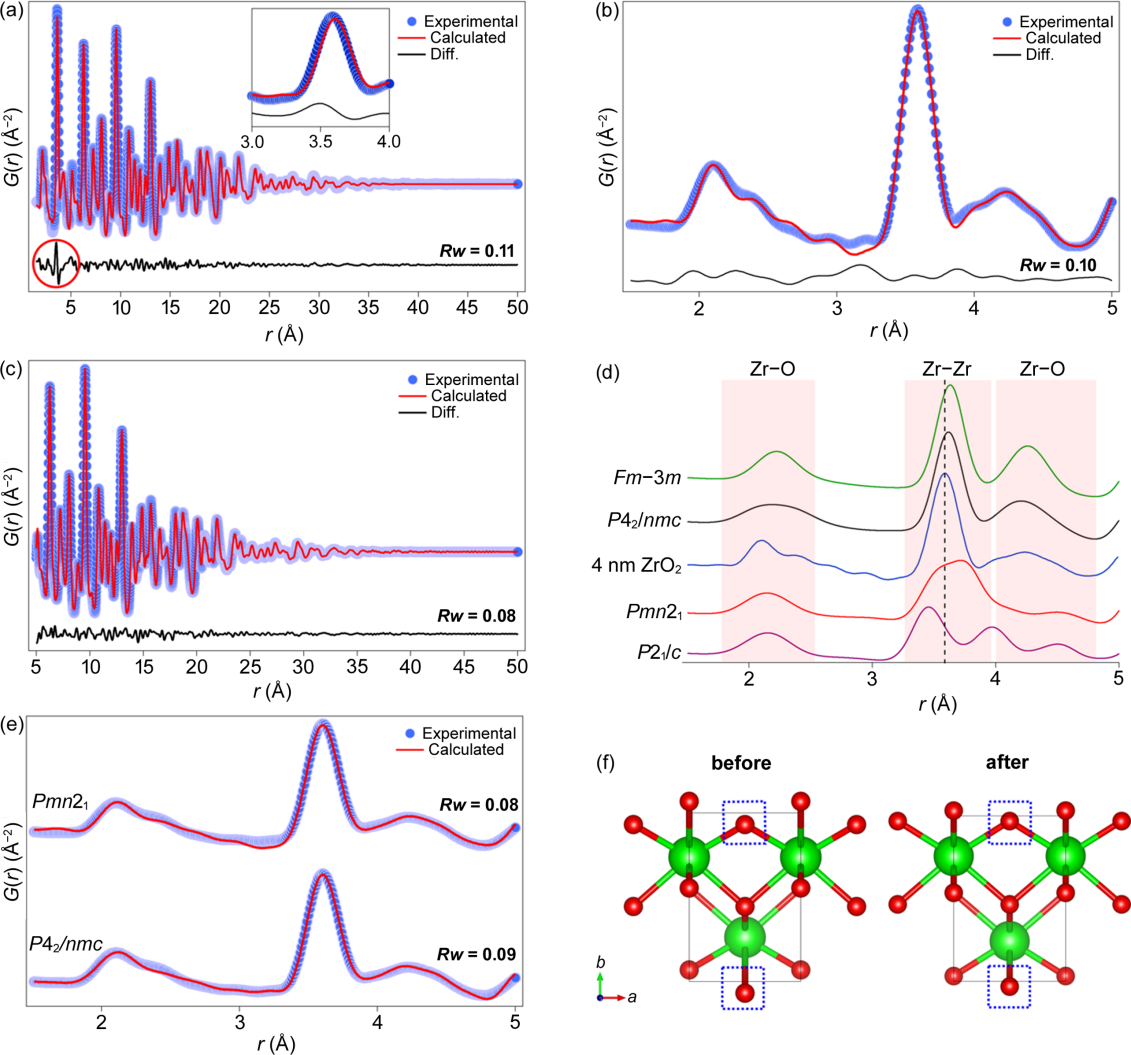
Experimental and calculated PDFs for 4 nm ZrO_2_ nanocrystals with the tetragonal (*P*4_2_*/nmc*) model for the ranges (*a*) 1.5–50 Å, (*b*) 1.5–5 Å and (*c*) 5–50 Å. (*d*) Comparison of the simulated PDFs for different ZrO_2_ polymorphs with the experimental data. The space groups of the polymorphs used are indicated. (*e*) Short-range fit obtained for 4 nm ZrO_2_ after relaxing the atomic positions of Zr by constraining the symmetry. (*f*) The unit cell of *Pmn*2_1_ before and after optimizing the Zr atom position.

X-ray total scattering with PDF analysis is a well known technique for the detailed structural analysis of nano­structured and disordered materials (Billinge & Levin, 2007 ▸). The analysis is particularly useful because it accounts for local distortions in the material, allowing the identification of disorders. ZrO_2_ nanocrystals (4 nm in diameter) were synthesized in trioctylphosphine oxide (TOPO) at 340°C, purified and subsequently analyzed with total scattering:
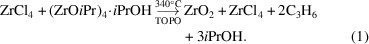
The experimental PDF was modeled with a tetragonal (*P*4_2_/*nmc*) phase, but a significant misfit was observed for the first Zr—Zr peak (3.59 Å). All other peaks showed agreement with the model [Fig. 1 ▸(*a*)]. Although the fit is quite good (*Rw* = 0.11), the mismatch observed for the first Zr—Zr peak (3.59 Å) could be attributed to minor variations in the local structure of the synthesized nanocrystals, compared with the average structure. Conventional powder diffraction techniques are limited in detecting such differences due to peak broadening caused by the small crystallite size (Egami & Billinge, 2012 ▸). The inset in Fig. 1 ▸(*a*) illustrates the source of the mismatch, indicating that it originates from an experimentally shorter Zr—Zr distance than predicted by the model, while the intensities of the two peaks are similar. A better fit is obtained by dividing the fitting range into two parts: 1.5–5 Å [Fig. 1 ▸(*b*)] and 5–50 Å [Fig. 1 ▸(*c*)]. The refinements were made by fixing the crystallite size to 40 Å. The fit parameters obtained for the 1.5–50 and 5–50 Å ranges show similar values, while those for the 1.5–5 Å range are different (Table S1 of the supporting information). An increase in the lattice parameter *a*, the isotropic displacement parameter (*U*_iso_) of oxygen and a decrease in the lattice parameter *b* are observed in the 1.5–5 Å range. The higher *U*_iso_ values for oxygen in the range 1.5–5 Å suggest the presence of local disorder, meaning that the local and average structures differ from each other.

In Fig. 1 ▸(*d*), short-range X-ray PDF simulations are shown for various ZrO_2_ phases, along with their deviation from the observed PDF of the synthesized ZrO_2_ nanocrystals. In the cubic phase (*Fm*3*m*) of ZrO_2_, only one type of short Zr—Zr spacing is observed at 3.63 Å due to its high symmetry. On the other hand, in the tetragonal phase (*P*4_2_/*nmc*), there are two types of pairs with equal contributions to the peak intensity, located at 3.59 and 3.62 Å. Although the peak positions are very close, a slightly broader peak is observed. Monoclinic (*P*2_1_/*c*) is the most distorted structure among the different ZrO_2_ phases, with even shorter Zr—Zr distances ranging from 3.3 to 4 Å. In this case, two broad peaks are observed. Several orthorhombic phases such as *Pmn*2_1_, *Pca*2_1_, *Pbca* and *Pnma* have been reported with average Zr—Zr distances that fall between those of the monoclinic and tetragonal phases (Ramprasad *et al.*, 2014 ▸). The Zr—Zr spacing observed in the experimental PDF of 4 nm nanocrystals does not match any known polymorphs and falls between the values for the tetragonal and monoclinic phases. The intensity of the second Zr—O peak (between 4 and 5 Å) is much lower in the experimental PDF than in the calculated tetragonal phase and multiple peak contributions are observed in the first Zr—O interaction [Fig. 1 ▸(*d*)].

By fitting the local range with various polymorphs, as shown in Fig. S1 and Table S2 of the supporting information, it was observed that the fits with *P*4_2_/*nmc* and *Pmn*2_1_ were more suitable in terms of peak positions and refined parameters (Table S2). However, the *U*_iso_ value of oxygen was higher in the *P*4_2_/*nmc* model. This suggests that the distortions present in the structure were compensated using a higher *U*_iso_ value, leading to a better fit agreement. Notably, the *U*_iso_ values obtained for *Pmn*2_1_ are relatively low, and the fit can be substantially improved by allowing additional freedom to the positions of the Zr atoms given that the symmetry is constrained [Fig. 1 ▸(*e*)]. The unit cell, before and after refinement of the Zr atom positions, is illustrated in Fig. 1 ▸(*f*), and the refined parameters are listed in Table S3. However, unlike *Pmn*2_1_, no such improvement was observed in the case of *P*4_2_/*nmc*. The findings suggest that the local structure can be described as a slightly distorted *Pmn*2_1_ phase which belongs to the non-centrosymmetric orthorhombic crystal system. However, the average structure remains assigned to the tetragonal phase. X-ray diffraction (XRD) patterns of *P*2_1_/*c*, *P*4_2_/*nmc* and *Pmn*2_1_ were simulated for the crystallite size of 4 nm (Fig. S2). The reflections of the *P*4_2_/*nmc* phase match best with the experimental results.

### 2.2. Size-dependent structure

**Figure 2:**
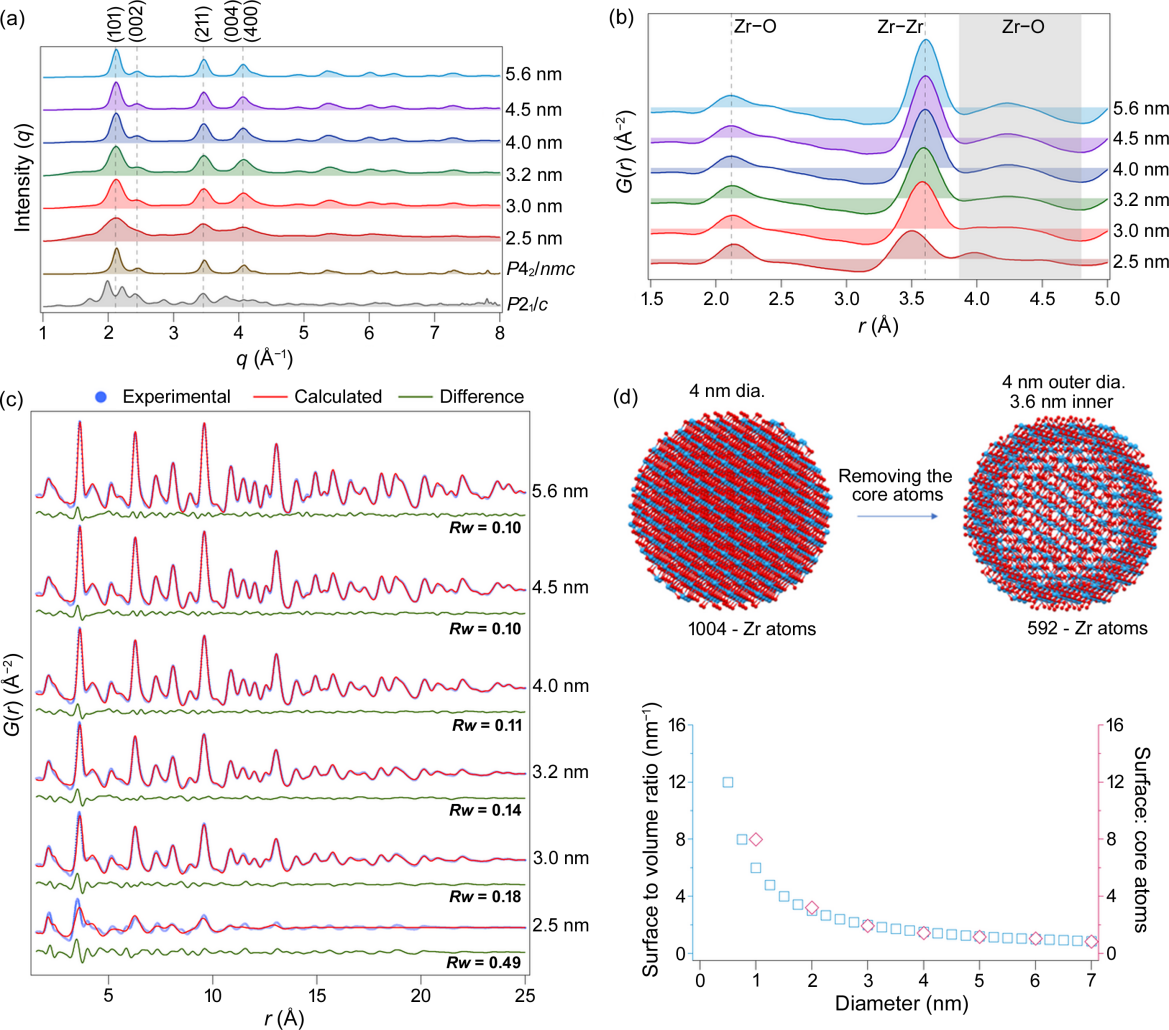
(*a*) XRD and (*b*) PDF for the ZrO_2_ nanocrystal size series. (*c*) Experimental and calculated PDFs for the ZrO_2_ nanocrystal size series fitted with *P*4_2_/*nmc*. The refined crystallite size (*p*_size_) and *Rw* value (goodness of fit) are indicated. Other refined parameters are given in Table S4. (*d*) Surface-to-volume ratio and surface-to-core atom ratio for ZrO_2_ nanocrystal size series. The intensities of the XRD patterns have been normalized for better visualization. The particle size was determined from the corresponding PDF refinements and is shown in the figure. In the calculation of the surface-to-atom ratio, a surface thickness of 0.4 nm was assumed and only Zr atoms were considered for quantification.

ZrO_2_ nanocrystals of various sizes (2.5–5.6 nm) are prepared following our previously established size-tuning methods. A comparison of nanocrystal sizes obtained from different techniques, including high-resolution transmission electron microscopy and small-angle X-ray scattering, was presented in our previous work (Pokratath *et al.*, 2022 ▸; Pokratath *et al.*, 2023 ▸). The XRD patterns of the resulting size series can be seen in Fig. 2 ▸(*a*), and they appear to be similar to the tetragonal phase (*P*4_2_/*nmc*) of ZrO_2_. Strong peak broadening is observed for the smaller nanocrystals where the 101 and 002 reflections overlap. The intensity of the 2.5 nm nanocrystal pattern has been magnified for clarity. This results in a higher baseline compared with the other samples. However, we emphasize that the elevated baseline should not be interpreted as an additional peak or structural feature. As the nanocrystal size increases, the peaks become sharper, but no significant changes in the structural features were observed. A distinct difference is visible in the corresponding signals when transformed to PDF, as shown in Fig. 2 ▸(*b*). A shift can be observed for the first Zr—Zr peak, which is correlated with the changes in the crystallite size. As the size increases, the peak position increases and shifts towards the position in the pure tetragonal phase (3.61 Å). The shift is more noticeable for smaller nanocrystals. Note that the second Zr—O peak [shaded region in Fig. 2 ▸(*b*)] for the 2.5 nm nanocrystal appears to have split into two, resembling the simulated pattern of the pure monoclinic phase (*P*2_1_/*c*) shown in Fig. 1 ▸(*d*). The particular pattern between 3.9 and 5 Å is not a consequence of the small nanocrystal size as shown by the calculated PDFs for different tetragonal crystallites (Fig. S3). However, since no clear traces of the monoclinic phase were observed with Rietveld analysis (Fig. S4), this pattern may be attributed to a highly distorted structure that incorporates elements of both the tetragonal and the monoclinic phases.

Refining the PDFs with the tetragonal phase (*P*4_2_/*nmc*) results in good fits for larger crystals, but the fit becomes increasingly poor for crystals smaller than 4 nm, as shown in Fig. 2 ▸(*c*). For the 2.5 nm nanocrystals, we explored the pure monoclinic phase as an alternative (see Fig. S5). The nonphysical refined *U*_iso_ values, coupled with the absence of any traces of a pure monoclinic phase in the Rietveld analysis, effectively rule out the transformation into pure monoclinic phase. When the size of the nanocrystals decreases, the proportion of surface atoms compared with those in the core increases. From Fig. 2 ▸(*d*), it can be seen that the ratio of surface to core atoms (assuming the surface thickness to be 4 Å) is approximately 1 when the diameter is 6 nm. As the diameter decreases below 6 nm, the surface atoms dominate over the core atoms. The presence of broken bonds on the terminating surface atoms causes a difference in the bond distance between the surface atom and its nearest neighbor in the core compared with the bulk. The PDFs shown in Fig. 2 ▸(*b*) suggest that the bond length between the Zr atom on the surface and its nearest neighbor may be shorter than average. This could explain the peak shift for very small nanocrystals [Fig. 2 ▸(*c*)]. Since we do not observe the peak shift in the simulated PDF of nanocrystals (Fig. S3) and the peak position shifts significantly for smaller crystallite sizes, the observed effect is attributed to the surface defects. Note that even the larger (5 nm) particles still feature the particular misfit that we previously attributed to the local orthorhombic structure. The surface provides an additional effect on the PDF.

To further investigate if the ligands on the surface have any effect on the surface defects, we performed PDF analysis before and after surface modification. Previously reported findings indicate that the synthesized ZrO_2_ nanocrystals are capped with TOPO and its decomposition products (De Keukeleere *et al.*, 2017 ▸). To eliminate the effect of the ligands from the analysis, we modified the surface by treating it with dilute HCl. Once the ligands exchanged, the surface chemistry altered and the nanocrystals were no longer colloidally stable, confirming the successful modification of the surface. We compared the PDF analysis of the surface-modified nanocrystals with the original ones, and no changes were detected in the peak position or refined parameters (Fig. S6 and Table S6). This points to the absence of any significant effect of ligands on the PDF.

### 2.3. Efforts to characterize the local dipole

Building on the structural analysis using the PDF, we observed that the material exhibits a non-centrosymmetric *Pmn*2_1_ polar phase at the local level. This structural characteristic is critical as it suggests the potential for ferroelectric behavior, highlighting the importance of understanding the structure–function relationship to fully harness the material’s functional capabilities. To explore this potential, we conducted switching spectroscopy piezoelectric force microscopy (SS-PFM) experiments – an effective technique that enables nanoscale mapping of switching parameters by acquiring the local PFM hysteresis loop – to investigate the ferroelectric properties of the ZrO_2_ nanocrystals (3 and 4 nm). We prepared ZrO_2_ nanocrystal-based thin films on a conducting electrode via spin coating (see experimental), which were subsequently annealed at 400°C to enhance the packing efficiency and minimize the ligand fraction. Fig. S7 displays the atomic force microscopy (AFM) images obtained before and following annealing. The spin-coat annealing cycle was repeated two times to improve the surface morphology.

**Figure 3:**
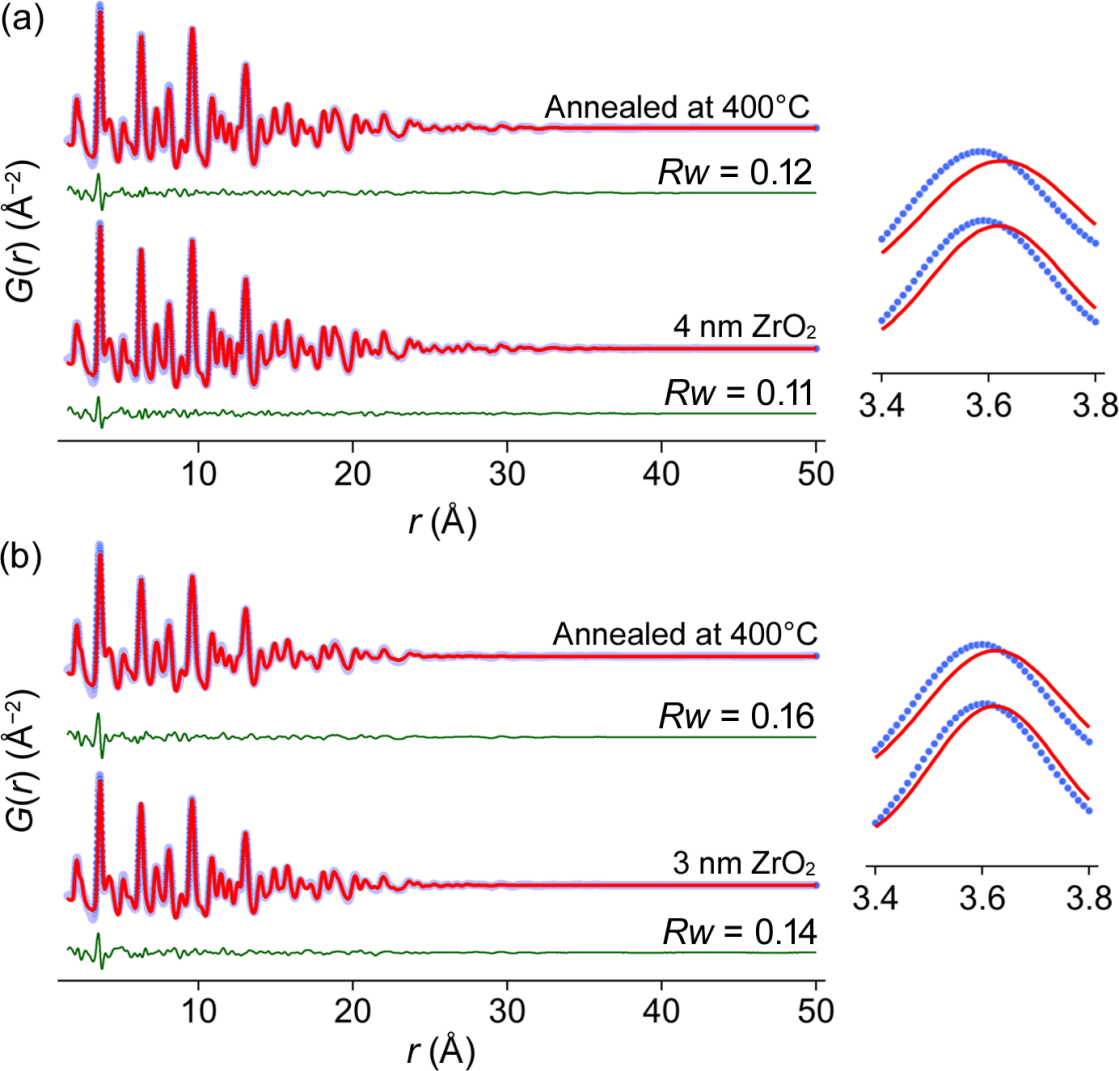
PDF analysis after heat treatment. The PDF of (*a*) 4 nm and (*b*) 3 nm nanocrystals after annealing at 400°C for 1 h in air. The misfit at the first Zr—Zr peak is magnified to improve visualization. The refined parameters can be found in Tables S7 and S8.

We first investigated the effect of the thermal treatment. After annealing the nanocrystals at 400°C for 1 h, we observed changes in the PDF of the nanocrystals [Figs. 3 ▸(*a*) and 3 ▸(*b*)]. The misfit for the first Zr—Zr peak is more pronounced, the crystallite size reduced by about 2 Å, and the *U*_iso_ values of zirconium and oxygen atoms increased (Tables S7–S8). The higher *U*_iso_ values indicate more disorder. Given that there is only a slight reduction in crystallite size and the XRD patterns appear similar (as shown in Fig. S8), it can be inferred that the observed effect is confined to the surface and that the overall structure remains the same. While the surface defects could be attributed to phosphate formation upon annealing as shown by Shaw *et al.* (2018 ▸), the exact structural changes that occurred at the surface are unclear. The absolute quantification of the contribution of surface defects to the peak shift is also not possible due to the presence of local distortions in the bulk of the crystal.

During SS-PFM measurements a DC voltage (*V*_DC_) sweep is applied in combination with a small AC voltage [*V*_AC_ cos(ω*t* + ϕ)] to probe the piezo-response (Balke *et al.*, 2015 ▸). The *V*_DC_ sweep consists of consecutive on-and-off switching, where the magnitude slowly increases and decreases over multiple cycles approximating a triangle waveform. This produces two types of loops: (i) when the *V*_DC_ is applied, called on-state; and (ii) when the *V*_DC_ is turned off, called off-state. For ferroelectric materials, the choice for a specific state loop depends on the research goals and experimental conditions. When the testing is done on a bare surface without a top electrode (like our case), it is recommended to use the off-state measurements as a way to reduce the electrostatic contribution to the PFM signal (Balke *et al.*, 2015 ▸). In contrast, on-state measurements allow us to avoid polarization relaxation between pulses when no DC bias is present (Alexe & Gruverman, 2004 ▸). Independent of the state loop measurement, ferroelectrics typically exhibit a butterfly-type amplitude hysteresis loop accompanied by changes in the phase signal, whereas in the case of antiferroelectrics, since there is no net polarization in the absence of an external field, the local antiferroelectric behavior is tested in the on-state loop. The results obtained for the thin film with 4 and 3 nm nanocrystals are shown in Fig. S9. In these measurements the frequency of one cycle was 500 mHz; each cycle started at 0 V and rectangular wave pulses with 90 ms on and 90 ms off were decremented to a final voltage of −8 V, upon which the wave pulses were incremented to 8 V, then decremented back up to 0 V, giving a full piezoresponse versus tip bias hysteresis loop. The on-state does not show the typical antiferroelectric loop, and the off-state presents a ferroelectric hysteresis loop; the amplitude signal as a function of bias exhibits the characteristic ‘butterfly’ shape with a finite value at zero bias and two local minima that coincide with a phase shift of almost 180° in the phase signal, which normally corresponds to the coercive field. To verify our findings, the measurements were repeated but this time with a frequency of 200 mHz (see Fig. S10). In this case, there was no ‘butterfly’ shape in the amplitude signal and an expected, but distorted, phase shift in the phase signal was observed. This inconsistency in the results could be related to electrostatic effects (Kim *et al.*, 2016 ▸), electrochemical strain (Jesse *et al.*, 2012 ▸; Adler, 2001 ▸; Morozovska *et al.*, 2010 ▸) or any other potential artifacts that are beyond the scope of this paper. Although SS-PFM can be used to characterize materials already known to be ferroelectric, it is unreliable as actual proof of ferroelectricity. Given also the lack of robustness in our measurements, we cannot conclude that the zirconia nanocrystals are ferroelectric. This does not come as a complete surprise since the average structure is the nonpolar tetragonal structure and only the local structure is described by a polar distortion.

## 3. Conclusions

In this study, we used X-ray PDF analysis to gain a deeper understanding of the local structure of ZrO_2_ nanocrystals. Our results reveal an intriguing orthorhombic distortion that is present regardless of the crystallite size. This distortion suggests the potential presence of an intermediate phase that may play a role in the observed switching behavior. While we did not observe direct evidence of ferroelectricity in layers of annealed nanocrystals, our findings highlight the importance of local structural distortions in influencing the material’s electrical properties. These results provide valuable insight into the relationship between the local atomic arrangement and macroscopic behaviors, offering a foundation for further exploration into the design of zirconia-based materials with enhanced electrical properties. Future work will focus on elucidating the precise mechanisms that connect the local structure to the electrical performance, with the goal of tailoring these materials for potential applications in ferroelectric and switching devices.

## 4. Experimental

### 4.1. Materials

ZrCl_4_ (99.9%), ZrBr_4_ (99%) and Zr(O*t*Bu)_4_ (99.9%) were purchased from Strem Chemicals and Zr(O*i*Pr)4·*i*PrOH (99.9%), toluene (99.5%) and acetone (99.8%) from Sigma–Aldrich and were used without further purification. Tri-*n*-octylphosphine oxide (99%) was bought from Strem chemicals and recrystallized according to Owen *et al.* (2008 ▸).

### 4.2. Nanocrystal synthesis

ZrO_2_ nanocrystals are synthesized according to our previously published procedure (De Keukeleere *et al.*, 2017 ▸) which was slightly different from the original procedure of Joo *et al.* (2003 ▸). Typical amounts were 7.5 g of recrystallized TOPO, Zr(O*i*Pr)4·*i*PrOH (0.387 g, 1.5 mmol) and ZrCl_4_ (0.349 g, 1.5 mmol). To synthesize smaller nanocrystals, either the reaction is stopped on the basis of the temporal evolution of nanocrystal size or precursor combinations with ZrBr_4_ or Zr(O*t*Bu)_4_ are used. Larger ZrO_2_ nanocrystals were produced using continuous injection of either Zr(O*i*Pr)4·*i*PrOH or Zr(O*t*Bu)_4_ into the reaction mixture (Pokratath *et al.*, 2022 ▸; Pokratath *et al.*, 2023 ▸).

### 4.3. Thin film preparation

Purified nanocrystals were dispersed in toluene at a concentration of 100 mg ml^−1^. The sample (50 µl) was spin-coated on a 12.5 × 12.5 mm indium tin oxide coated substrate at 1000 rpm for 30 s, and then the substrate was moved to a hot plate at 400°C. After 1 h, the substrate was taken out and allowed to cool to room temperature.

### 4.4. Synchrotron X-ray total scattering experiments

Samples were prepared in 1 mm polyamide Kapton tubes and were measured at beamline 11-ID-B at the Advanced Photon Source, Argonne National Laboratory, USA. X-ray total scattering data were collected at room temperature in rapid-acquisition mode, using a PerkinElmer digital X-ray flat-panel amorphous silicon detector (2048 × 2048 pixels and 200 × 200 µm pixel size) with a sample-to-detector distance of 180 mm (11-ID-B). The incident wavelength of the X-rays was λ = 0.2110 Å (11-ID-B). Calibration of the experimental setup was performed using an Ni standard.

### 4.5. Analysis of synchrotron X-ray total scattering data

Raw 2D data were corrected for geometrical effects and polarization and then azimuthally integrated to produce 1D scattering intensities versus the magnitude of the momentum transfer *Q* (where *Q* = 4π sin θ / λ for elastic scattering) using *pyFAI* and *xpdtools* (Ashiotis *et al.*, 2015 ▸; Wright & Zhou, 2017 ▸). The program *xPDFsuite* with *PDFgetX3* was used to perform the background subtraction; further corrections and normalization to obtain the reduced total scattering structure function *F*(*Q*); and Fourier transformation to obtain the PDF, *G*(*r*) (Juhás *et al.*, 2013 ▸; Yang *et al.*, 2014 ▸). For data reduction, the following parameters were used after proper background subtraction: *Q*_min_ = 0.8 Å^−1^, *Q*_max_ = 22 Å^−1^ and *R*_poly_ = 0.9 Å. Modeling and fitting were carried out using *Diffpy-CMI* (Juhás *et al.*, 2015 ▸).

### 4.6. Surface topology

The morphology of ZrO_2_ coatings was investigated using AFM in air (NanoWizard Ultra, JPK Instruments, Bruker, USA) in intermittent contact mode. Commercially available aluminium reflex-coated cantilever Tap150Al-G (nominal resonant frequency 150 kHz, spring constant 5 N m^−1^) was used for imaging all the samples in air. Micrographs were collected at a drive frequency of 176 kHz with a line rate of 0.225 Hz. Data were subsequently analyzed with the *JPK* data analysis software.

### 4.7. SS-PFM measurements

SS-PFM was performed using the same setup as PFM as described by Jesse *et al.* (2006 ▸). A DC bias (*V*_DC_) is applied to the AFM tip in addition to the AC probing voltage (*V*_AC_), and the bottom electrode is grounded. The PFM phase and amplitude are then recorded while the DC bias is ramped stepwise, going back to zero bias after each step. This produces two measurements: one as a function of the bias applied at the time of the acquisition (‘on’), and one as a function of the bias applied just before the acquisition (‘off’). To avoid the effects of electrostatic interactions between tip and sample, only the results of the ‘off’ state are used. Amplitude and phase signals were recorded using an Asylum Research MFP-3D AFM operating in dual resonance tracking (DART) mode, using Nanosensores PPP-EFM cantilevers with a resonance frequency of 75 kHz and a spring constant of 2.8 N m^−1^.

## Supplementary Material

Supporting figures and tables. DOI: 10.1107/S1600576725001761/vb5079sup1.pdf
