# Supplementary material for: Local orthorhombic phase in zirconium oxide nanocrystals: insights from X-ray pair distribution function analysis
Source: J Appl Crystallogr. 2025 Apr 4;58(Pt 3):688–95. doi: 10.1107/S1600576725001761 (PMC12135986; doi:10.1107/S1600576725001761)
Supplement: Supplementary file 1 [file j-58-00688-sup1.pdf]

JOURNAL OF APPLIED CRYSTALLOGRAPHY

Volume 58 (2025)

Supporting information for article:

Local orthorhombic phase in zirconium oxide nanocrystals: insights from X-ray pair distribution function analysis

Rohan Pokratath, Kumara Cordero-Edwards, Maryame Bina, Simon J. L. Billinge and Jonathan De Roo

1 Figures

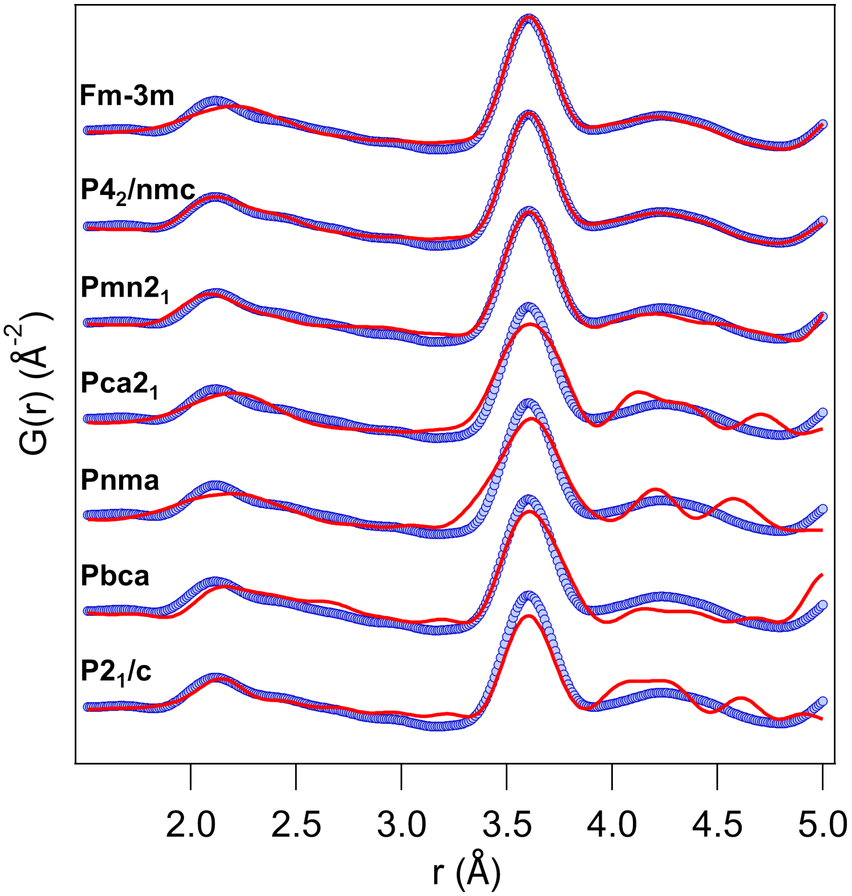

Figure S 1 | Fitting the local structure of 4 nm ZrO_2 with various models. The spacegroups are indicated and the refined parameters are given in Table S2.

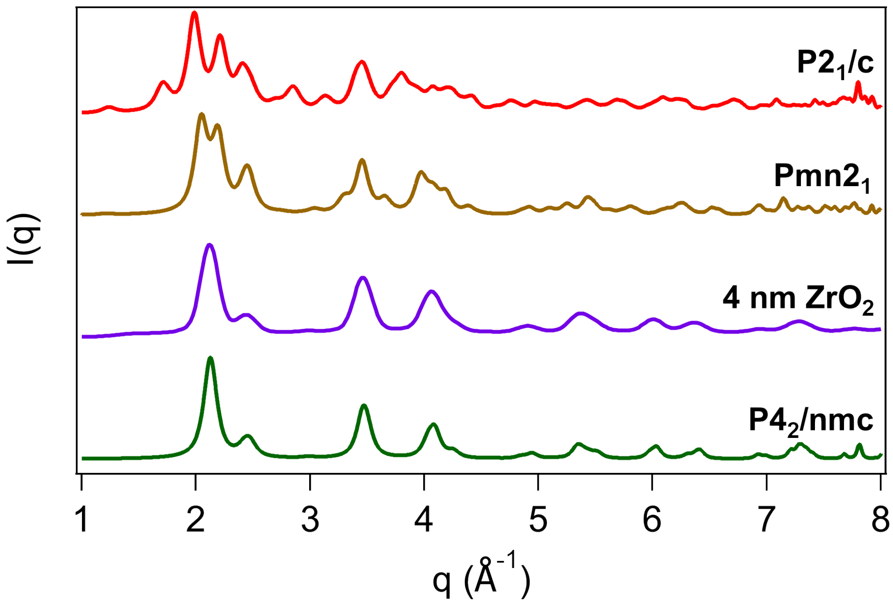

Figure S 2 | XRD pattern for average structure. Comparison of XRD pattern of 4 nm ZrO_2 with simulated patterns of P4$_2$/nmc, Pmn2$_1$, and P2$_1$/c. The patterns are simulated with an FWHM value of 2 which is calculated for the crystallite size 4 nm for the peak at 2.1 Å^{-1}.

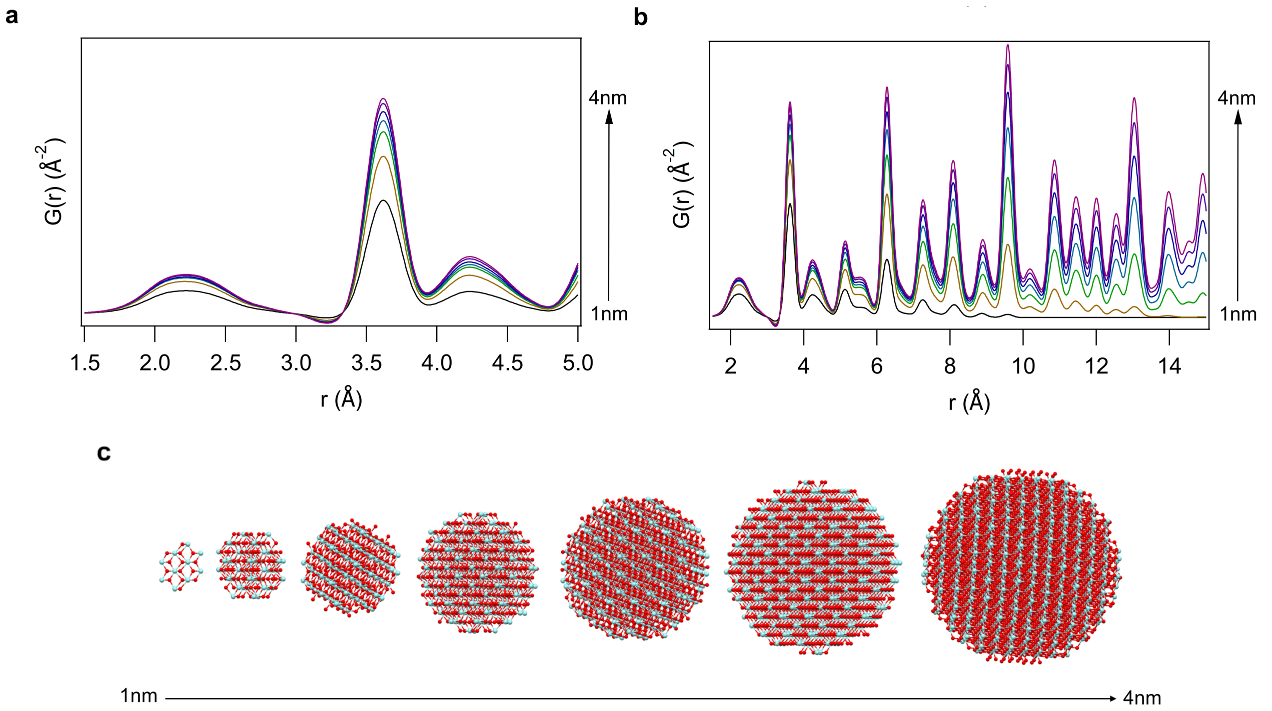

Figure S 3 | Simulated PDF of ZrO_2 size series. a Simulated PDF of pure tetragonal spherical nanocrystals, utilizing discrete models with sizes ranging from 1 nm to 4 nm. **b** The same simulated PDFs with a higher r-range. **c** The models used for the simulations.

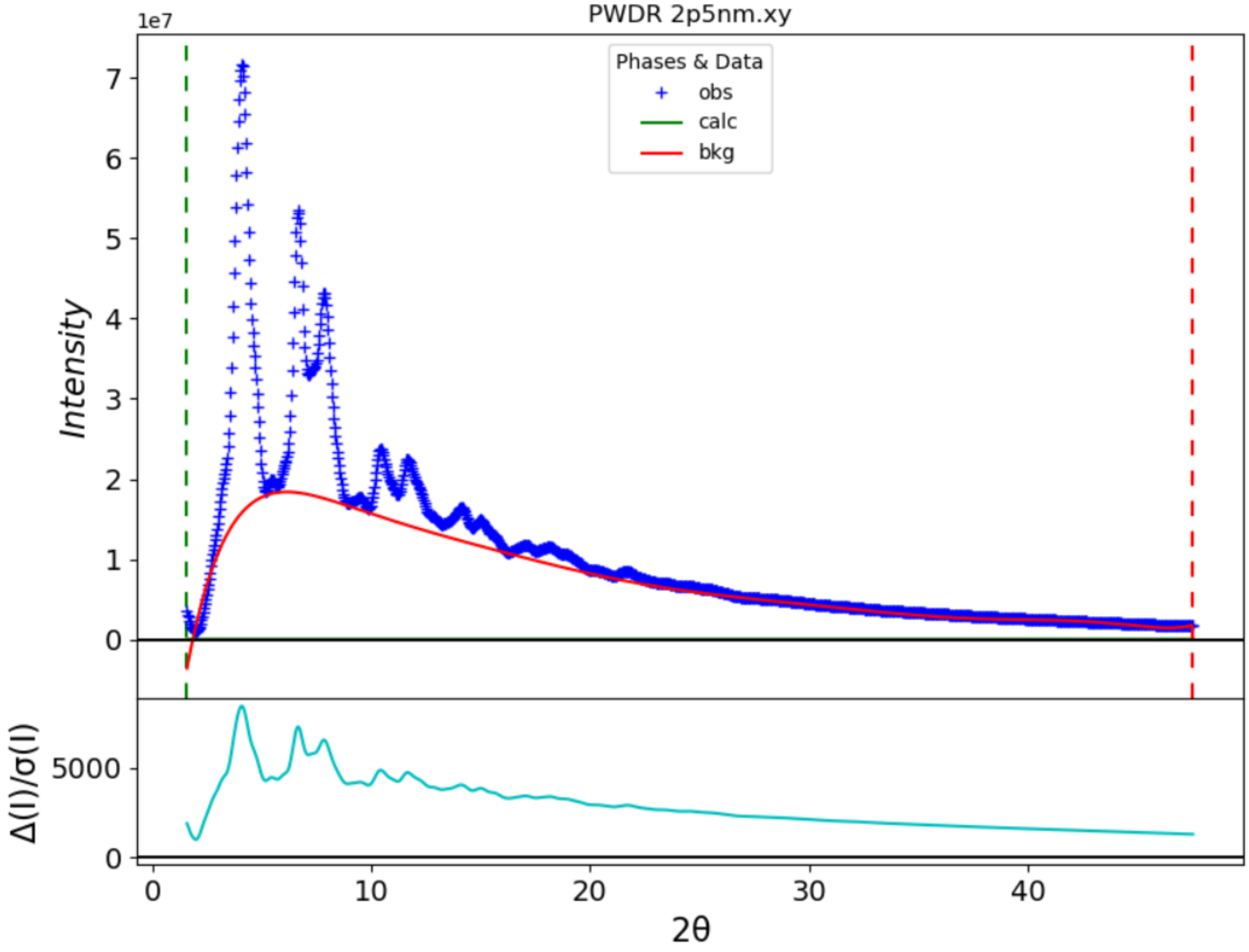

Figure S 4 | Rietveld Refinement of the XRD Pattern for 2.5 nm Nanoparticles. The refinement, performed with a monoclinic phase model, shows no detectable traces of the monoclinic phase in the XRD data. The refinement was performed using the GSAS-II software.

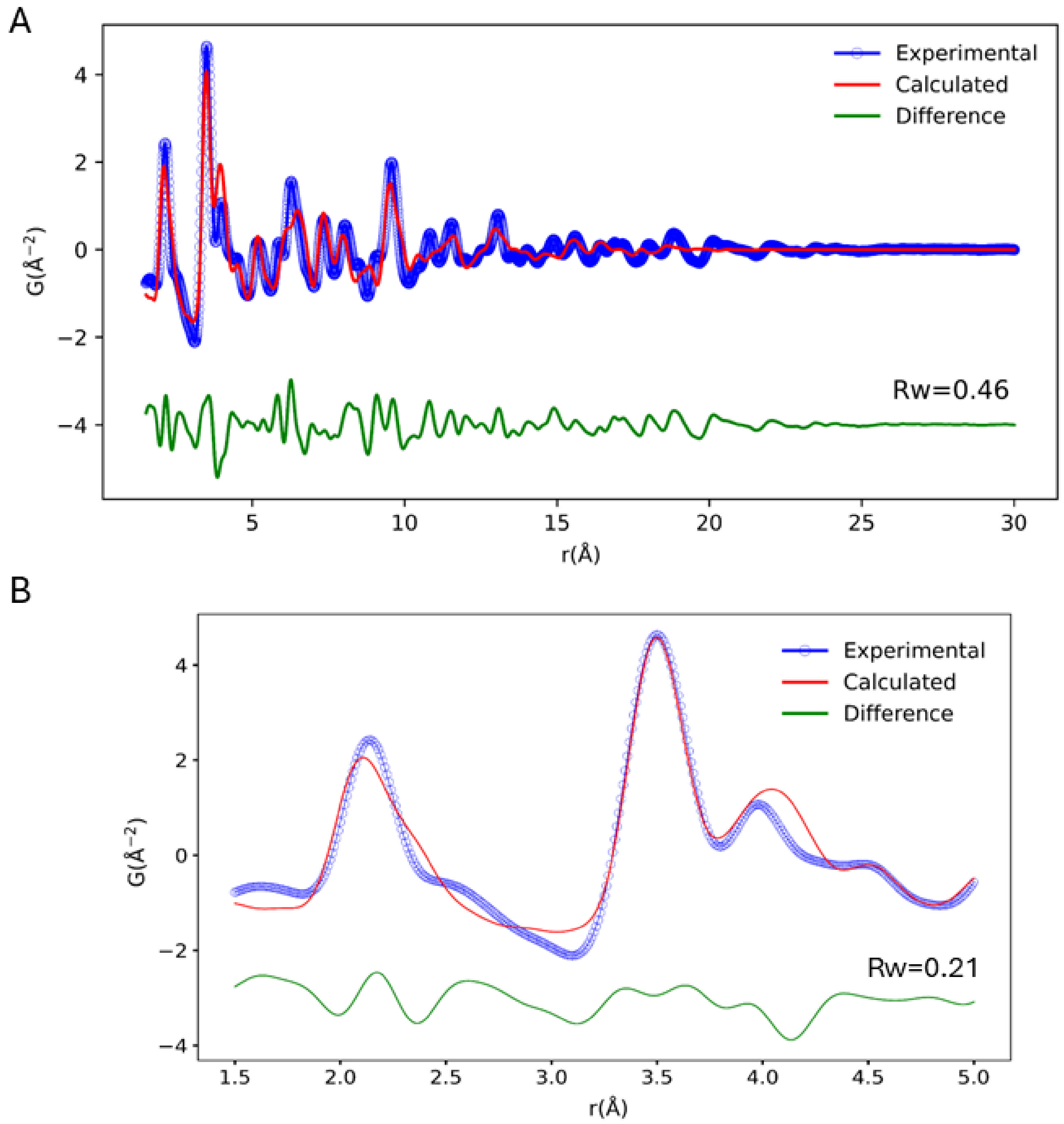

Figure S 5 | The PDF fit for 2.5 nm nanocrystal with pure monoclinic phase The PDF fit for 2.5 nm nanocrystal with pure monoclinic phase ($P2_1/c$) for (A) 1.5 - 30 Å and (B) 1.5 - 5 Å range. Refined parameters are indicated in Table S5.

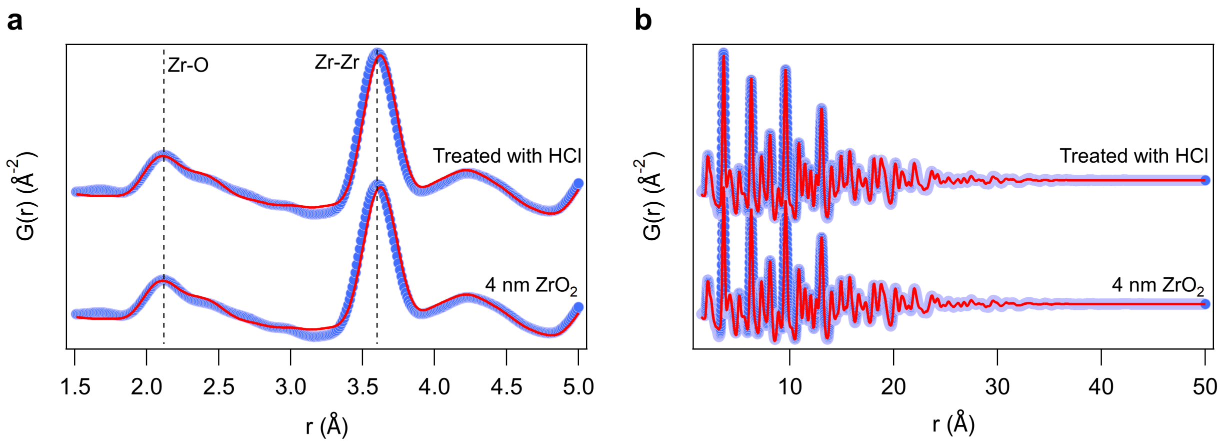

Figure S 6 | PDF analysis after surface treatment. a-b The PDF of 4 nm nanocrystals before and after modifying the surface with HCl.

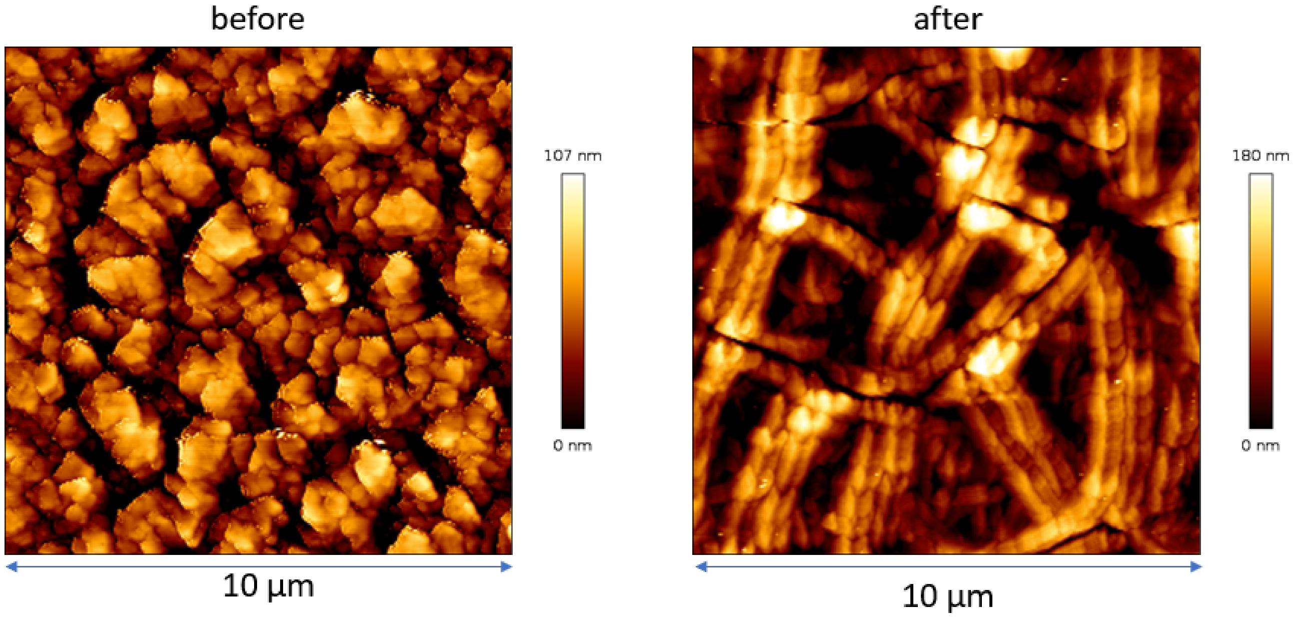

Figure S 7 | AFM images. The AFM images of thin film made by spin coating ZrO_2 nanoparticle solution before and after annealing at 400°C for 1 hour.

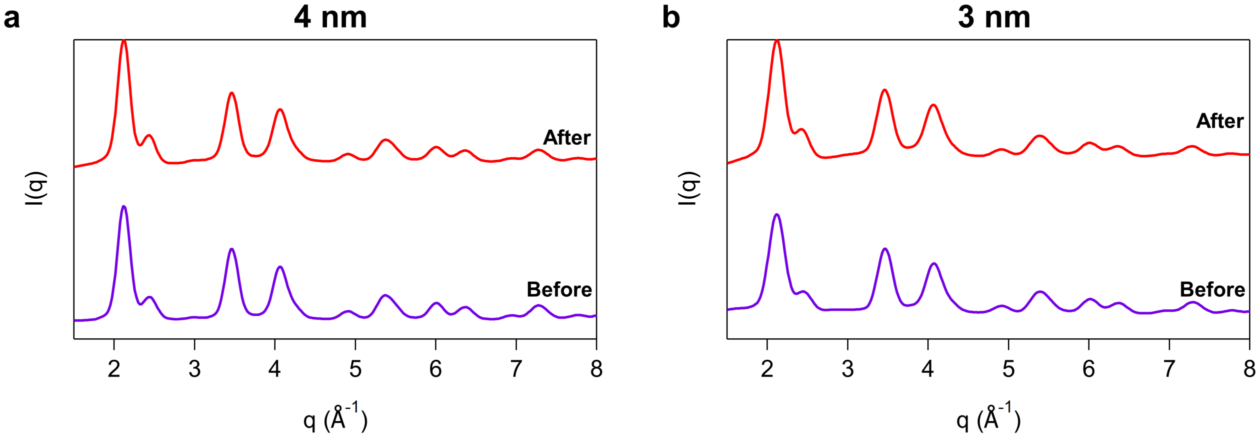

Figure S 8 | XRD after heat treatment. The XRD pattern of **(a)** 4 nm and **(b)** 3 nm nanocrystals before and after annealing at 400 °C for 1 hour.

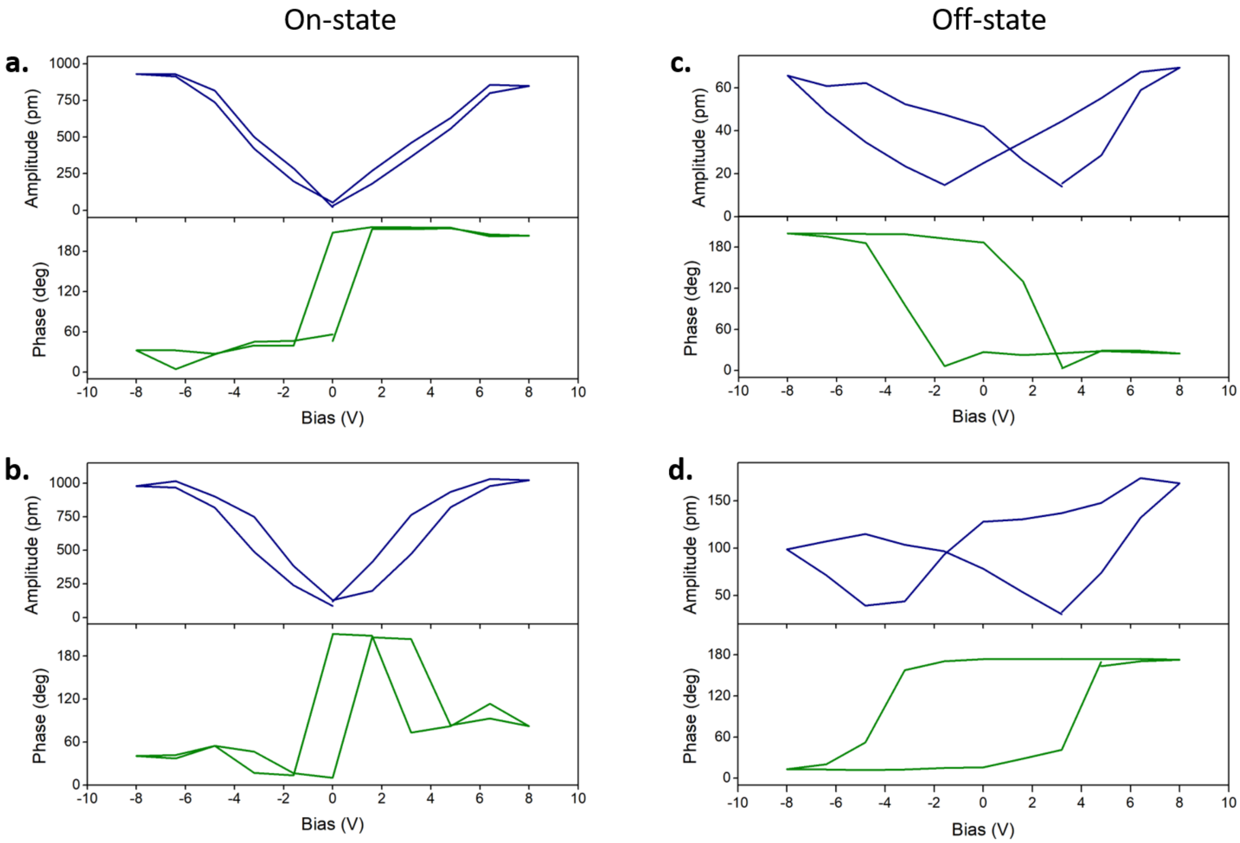

Figure S 9 | Hysteresis loop at 500 mHz for ZrO_2 nanocrystals based thin films. a-b On-state and **c-d** off-state hysteresis loop for a 4 nm and 3 nm ZrO_2 nanocrystal thin film.

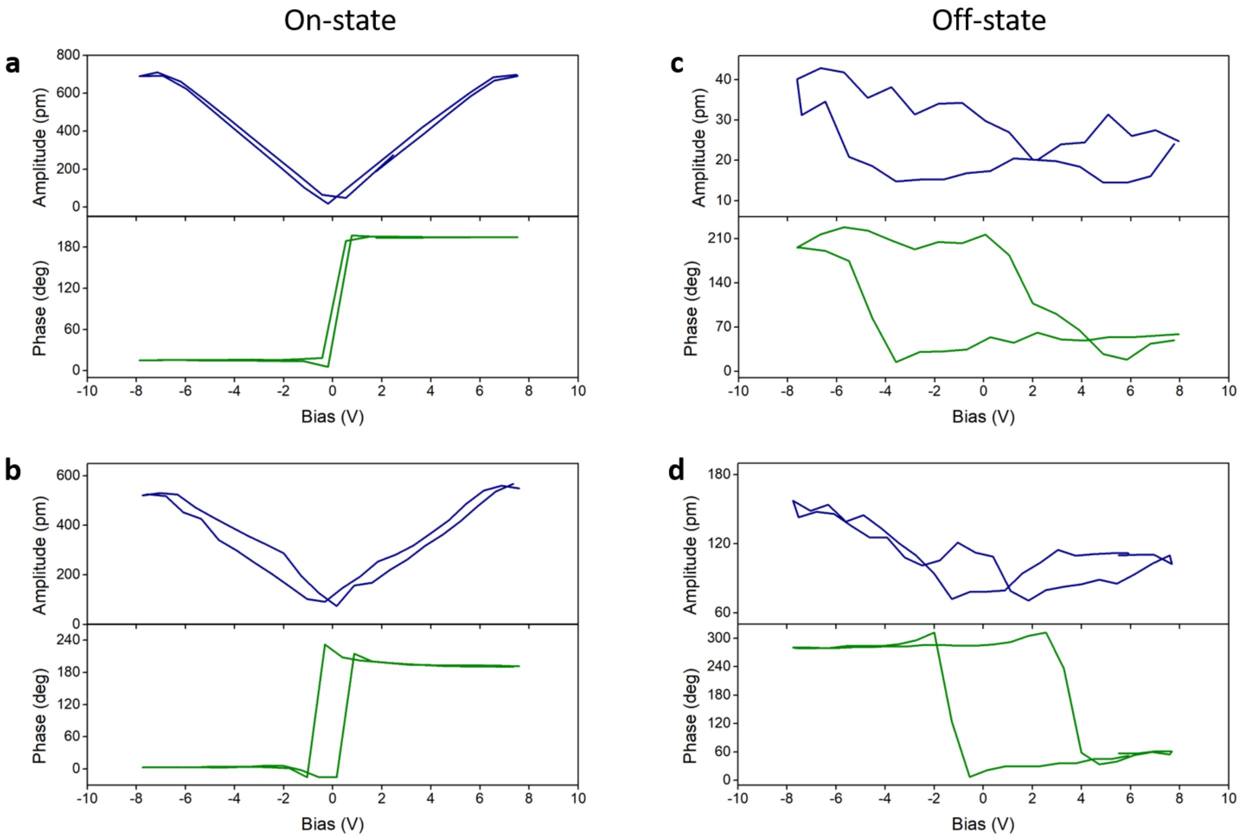

Figure S 10 | Hysteresis loop at 200 mHz for ZrO_2 nanocrystals based thin films. **(a)**-**(b)** On-state and **(c)**-**(d)** off-state hysteresis loop for a 4 nm and 3 nm ZrO_2 nanocrystal thin film.

2 Tables

Fit range	scale	psize[Å]	a[Å]	c[Å]	Uiso Zr[$Å^2$]	Uiso O[$Å^2$]	*Rw*
**1.5-50** Å	0.76	40±1	3.60	5.18	0.008	0.049	0.11
**1.5-5** Å	0.74	40(fixed)	3.64	4.99	0.007	0.062	0.10
**5-50** Å	0.76	40(fixed)	3.60	5.18	0.008	0.048	0.08

Table S 1 | Refined parameters after fitting 4 nm ZrO_2 in various ranges.

Spacegroup	scale	psize[Å]	a[Å]	b[Å]	beta[°]	c[Å]	Uiso Zr[$Å^2$]	Uiso O[$Å^2$]	*Rw*
**Fm-3m**	0.74	40(fixed)	5.09	-	-	-	0.007	0.080	0.13
**$P4_2$/nmc**	0.74	40(fixed)	3.64	-	-	4.99	0.007	0.062	0.10
**$Pmn2_1$**	0.71	40(fixed)	3.62	5.09	-	3.60	0.004	0.023	0.16
**$Pca2_1$**	0.95	40(fixed)	4.70	5.35	-	5.55	0.002	0.007	0.30
**Pnma**	0.74	40(fixed)	3.62	5.99	-	6.18	0.002	0.034	0.35
**Pbca**	0.99	40(fixed)	5.31	5.71	-	9.18	0.004	0.110	0.35
**$P2_1$/c**	1	40(fixed)	5.21	5.58	96.60	5.54	0.002	0.851	0.33

Table S 2 | Refined parameters after fitting the local structure of 4 nm ZrO_2 with various structures.

Spacegroup	scale	psize[Å]	a[Å]	b[Å]	c[Å]	Uiso Zr[$Å^2$]	Uiso O[$Å^2$]	*Rw*
**$P4_2$/nmc**	0.74	40(fixed)	3.64	-	4.98	0.007	0.058	0.09
**$Pmn2_1$**	0.74	40(fixed)	3.57	5.24	3.54	0.005	0.027	0.08

Table S 3 | Refined parameters after fitting 4 nm ZrO_2 for the range 1.5 -5 Åusing $P4_2$/nmc and $Pmn2_1$. In addition, Zr atom positions are refined maintaining the symmetry of the spacegroup.

	scale	psize[Å]	a[Å]	c[Å]	Uiso Zr[$Å^2$]	Uiso O[$Å^2$]	*Rw*
**5.6 nm**	0.78	56±2	3.60	5.18	0.007	0.043	0.10
**4.5 nm**	0.77	45±1	3.60	5.18	0.008	0.049	0.10
**4.0 nm**	0.76	40±1	3.60	5.18	0.008	0.049	0.11
**3.2 nm**	0.73	32±1	3.60	5.17	0.010	0.049	0.14
**3.0 nm**	0.72	30±1	3.60	5.16	0.011	0.044	0.18
**2.5 nm**	0.65	25±1	3.58	5.12	0.025	0.026	0.49

Table S 4 | Refined parameters after fitting ZrO_2 size series using $P4_2$/nmc model.

Range	scale	psize[Å]	a[Å]	b[Å]	c[Å]	beta[°]	Uiso Zr[$Å^2$]	Uiso O[$Å^2$]	*Rw*
**1.5-30 Å**	0.87	23±3	5.20	5.15	5.52	95.13	0.011	0.108	0.46
**1.5-5 Å**	0.84	23(fixed)	5.07	5.37	5.43	97.35	0.009	0.147	0.21

Table S 5 | Refined parameters after fitting 2.5 nm ZrO_2 with monoclinic phase ($P2_1$/c).

	scale	psize[Å]	a[Å]	c[Å]	Uiso Zr[$Å^2$]	Uiso O[$Å^2$]	*Rw*
**Before**	0.76	40±1	3.60	5.18	0.008	0.049	0.11
**After**	0.80	40±1	3.60	5.18	0.008	0.049	0.10

Table S 6 | Refined parameters after fitting 4 nm ZrO_2 before and after surface treatment with HCl.

	scale	psize[Å]	a[Å]	c[Å]	Uiso Zr[$Å^2$]	Uiso O[$Å^2$]	*Rw*
**Before**	0.76	40±1	3.60	5.18	0.008	0.049	0.11
**After**	0.85	38±1	3.60	5.18	0.011	0.054	0.12

Table S 7 | Refined parameters after fitting 4 nm ZrO_2 before and after annealing at 400 °C for 1 hour.

	scale	psize[Å]	a[Å]	c[Å]	Uiso Zr[$Å^2$]	Uiso O[$Å^2$]	*Rw*
**Before**	0.71	32±1	3.60	5.17	0.010	0.048	0.14
**After**	0.86	31±1	3.60	5.17	0.014	0.054	0.16

Table S 8 | Refined parameters after fitting 3 nm ZrO_2 before and after annealing at 400 °C for 1 hour.
